# EORTC-1607-GITCG/ABC-09: Open-label first-line, single-arm phase II study of CisGem combined with pembrolizumab in patients with advanced or metastatic biliary tract cancer

**DOI:** 10.1016/j.tranon.2026.103003

**Published:** 2026-08-31

**Authors:** Angela Lamarca, Annett Maderer, Daniel C. Wagner, Mairéad G. McNamara, John A. Bridgewater, Arvind Arora, Teresa Macarulla, Jorge Adeva, Richard A. Hubner, Florian Lordick, Friedrich Foerster, Arndt Weinmann, Peter R. Galle, Bryan Leurquin, Sandrine I. Marreaud, Murielle E. Mauer, Juan W. Valle, Markus Moehler

**Affiliations:** a Department of Medical Oncology – OncoHealth Institute, Fundación Jimenez Diaz University Hospital, Health Research Institute-Fundación Jimenez Diaz University Hospital (IIS-FJD), Universidad Autónoma de Madrid (UAM), Madrid, Spain; b Department of Medical Oncology, The Christie NHS Foundation Trust, Manchester, UK; c Department of internal medicine I, University Medical Center of the Johannes Gutenberg University Mainz, Mainz, Germany; d Department of Pathology, University Medical Center of the Johannes Gutenberg University Mainz, Mainz, Germany; e Division of Cancer Sciences, University of Manchester, Manchester, UK; f University College London Cancer Institute, University College London, London, UK; g Department of Medical Oncology, University Hospital of Nottingham NHS Trust, Nottingham, UK; h Department of Medical Oncology, Vall d'Hebrón Institute of Oncology, Vall d'Hebrón University Hospital, Barcelona, Spain; i Department of Medical Oncology, Hospital Universitario 12 de Octubre, Madrid, Spain; j Department of Oncology, Gastroenterology, Hepatology and Pulmonology, University of Leipzig Medical Center, Leipzig, Germany; k The European Organisation for Research and Treatment of Cancer (EORTC) Headquarters, Brussels, Belgium; l Cholangiocarcinoma Foundation, Herriman, UT, USA

**Keywords:** Advanced biliary tract cancer, Pembrolizumab, Immunotherapy, Immune environment, MDSCs, CD4/PD1+ and CD8/PD1+ Tcells, Neutrophil-lymphocyte-ratio

## Abstract

**Introduction:**

Immunotherapy recently approved with first-line (1L) chemotherapy for advanced biliary tract cancer (aBTC). Translational research is warranted to identify biomarkers for efficacy.

**Methods:**

This prospective multicenter non-randomised phase-II study recruited 50 treatment-naïve proven aBTC patients for cisplatin and gemcitabine with pembrolizumab until disease progression or unacceptable toxicity. Primary endpoint was progression-free survival at 6 months (6mPFS, RECIST v.1.1). Secondary endpoints were overall survival (OS), radiological responses and safety. Exploratory translational research was to identify predictive biomarkers. Tumour tissue and blood were collected for MMR, PD-1/PD-L1 in T-cells, monocytes and myeloid derived suppressor cells (MDSCs).

**Results:**

Fifty patients were 38% male, aged 63 (35–84) years. 6mPFS was 61.2% (80% CI 51.7–69.5) with mPFS 8.3 months (95% CI 5.6–10.6); ORR 40.8% (95% CI 27.0–55.8). Med. OS was 13.4 months (95% CI 8.3–20.5) with 24-months-OS 28.4% (95% CI 16.6–41.4). Higher PD-L1 expression correlated with improved PFS (HR=0.59; 95% CI: 0.26–1.37) and OS (HR=0.68; 95% CI: 0.28–1.64). Responders showed higher CD8/Treg ratio than non-responders (26.2% vs 21.2%) and lower CD4/PD1+ and CD8/PD1+ T-cells (33.5% vs 41.5% and 26.4% vs 44.7%) respectively. During treatment, profiling decreased in CD4/PD1+ and CD8/PD1+ Tcells (36.0% vs 22.2% and 41.6% vs 21.0%) respectively. MDSCs increased in non-responders. Responders, CPS-positive and low-stage patients showed normal Neutrophil-Lymphocyte-Ratios.

**Conclusion:**

Pembrolizumab plus CisGem showed consistent efficacy to prior phase-III trials. Biomarkers suggest baseline immune landscape influences responses, T-cell exhaustion and immunosuppressive myeloid expansion. Immune phenotyping supports patient selection and further biomarker-driven trials in BTC.

## Highlights

• Higher ORR (40.8%) than in comparable phase III studies.
• Immune-checkpoint inhibition and CisGem showed mPFS of 8.3 and mOS of 13.4 months.
• Early immune contexture (CD8/Treg) was more relevant than on-treatment changes.
• Immunosuppressive shift was seen in Non-R by increase in MDSCs and clas. monocytes.
• Responders and CPS+ patients are characterized by a normal NLR.

## Introduction

Biliary tract cancer (BTC), including cholangiocarcinoma (CCA) and gallbladder cancer (GBC), are aggressive malignancies arising from the biliary epithelium [1]. Unfortunately, most BTC cases present at an advanced stage, with limited survival despite active therapy. Cisplatin and gemcitabine (CisGem) remain the standard chemotherapy backbone for treatment of advanced BTC, based on the ABC-02 clinical trial [2]. Over the last few years, addition of check-point inhibitors either with durvalumab [3] or pembrolizumab [4] have shown an improvement in terms of overall survival (OS) and are now considered standard of care for all patients with advanced BTC (aBTC). In the absence of targetable alterations, treatment upon progression to first-line is limited to FOLFOX [5] or irinotecan-based chemotherapy [6]. For patients with targetable alterations such as isocitrate dehydrogenase-1 (IDH1) mutations or fibroblast growth factor receprot-2 (FGFR2) fusions, treatment options include ivosidenib [7], pemigatinib [8] or futibatinib [9]. Zanidatamab has been recently approved for use in patients with Human Epidermal Growth Factor Receptor-2 (HER2neu) amplification [10]. Many other targeted therapies are under evaluation [1].

Despite these advances, the median overall survival (mOS) for patients diagnosed with aBTC remains limited (around 13 months) and an area of unmet need. Late diagnosis remains an issue, due to presenting symptoms that are non-specific and challenging to identify. On the other hand, a significant proportion of patients diagnosed in advanced stages are not fit for therapy [11]. While efforts are being done to improve these issues, improvements of therapies in the first-line therapies are a must, especially for the population of patients without targetable alterations.

In this regard, the addition of immunotherapy to chemotherapy has been one of the main advances.

The TOPAZ-1 trial [3,12] randomized 685 patients with aBTC to receive durvalumab plus CisGem (n = 341) or placebo with CisGem (n = 344). The primary endpoint was OS. The mOS was 12.8 months with durvalumab vs 11.5 months with placebo (HR 0.80 (95% CI, 0.66–0.97; p = 0.021), independent of PD-L1 expression. Progression-free survival (PFS) was longer in the durvalumab arm: 7.2 vs 5.7 months (HR 0.75 (95% CI 0.63–0.89); p-value 0.001). Overall response rate (ORR) was 26.7% vs 18.7%, respectively. This clinical trial was followed by the KEYNOTE-966 [4], similar study design that included pembrolizumab/placebo as the checkpoint inhibitor to be tested. The KEYNOTE-966 randomized 1069 patients with aBTC to pembrolizumab plus CisGem (n = 533) or placebo plus CisGem (n = 536). The study met its primary endpoint (OS), the mOS was 12.7 months with pembrolizumab vs 10.9 months with placebo (HR 0.83; 95% CI, 0.72–0.95; p = 0.0034), regardless of PD-L1 status. The median progression-free survival (mPFS) (6.5 vs 5.6 months (HR 0.86 (95% CI 0-75-1.00); p-value 0.023, did not meet prespecified threshold for statistical significance) and ORR (29%vs 29%) were similar in both arms. Grade ≥3 treatment-related adverse events occurred in 69.1% (pembrolizumab) vs 69.0% (placebo). In both studies, the toxicity profile was favourable, with no major concerns identified by the addition of the checkpoint inhibitor to the chemotherapy.

One of the main limitations of these two studies was the inability to identify predictive biomarkers for immunotherapy success. While microsatellite instability (MSI) high and mismatch repair (MMR) deficiency are very rare events in BTC (both under 5% of this patient population) [13], other factors may explain the activity of immunotherapy in these tumours. In this regard, chronic inflammation, as seen in BTC associated with primary sclerosing cholangitis, may enhance response to checkpoint inhibitors [14]. These two clinical trials demonstrated little/no role for PD-L1 as a predictive biomarker. The benefit from durvalumab in addition to chemotherapy was similar, regardless of PD-L1 expression [3]. For patients with Tumour Area Positivity (TAP) ≥1% (representing 60.9% of patients in the durvalumab arm and 67.3% in the placebo arm) the OS HR achieved was 0.79 (95% CI 0.61–1.00); similar to the one reported for patients with TAP<1% (representing 55.3% of patients in the durvalumab arm and 64.1% in the placebo arm (HR 0.86 (95% CI 0.60–1.23)). Similar findings were reported for pembrolizumab [4]: HR of 0.84 (95% CI 0.62–1.14) and HR of 0.85 (95% CI 0.72–1.00) for patients with PD-L1 combined positive score (CPS) of <1 (represented 76.1% of patients in the pembrolizumab arm and 79.1% in the placebo arm) and ≥1 (represented 79.1% of patients in the pembrolizumab arm and 84.7% in the placebo arm), respectively. Thus, immunotherapy is offered to all patients, in the absence of a contraindication for checkpoint inhibitors. However, translational research in this area is much needed. First to identify patients who may benefit most, and second, to identify potential mechanism of resistance that could inform on the potential role of immunotherapy in the second line setting for selected patients.

Taken together, these pivotal randomised studies have fully established the role of checkpoint inhibitors as part of first-line therapy for aBTC. However, the evolving landscape of immunotherapy in this setting highlights ongoing challenges, particularly in identifying which patients derive the greatest benefit. The search for robust biomarkers remains a priority, as current selection strategies rely largely on clinical exclusion rather than molecular prediction.

The EORTC-1607-GITCG/ABC-09 clinical trial reported here, represents an effort towards biomarker discovery in the setting of patients treated with CisGem combined with pembrolizumab for aBTC.

## Material and methods

### Study design and patient population

The EORTC-1607-GITCG/ABC-09 clinical trial was an open-label first-line, single-arm phase II study of CisGem combined with pembrolizumab in patients with locally advanced or metastatic BTC conducted by the EORTC Gastro-intestinal Tract Cancer group (GITCG) in collaboration with the Advanced Biliary Tract Cancer (ABC) group.

Eligible patients had histologically confirmed BTC, including intrahepatic (iCCA), distal (dCCA) and hilar (hCCA) cholangiocarcinoma or gallbladder cancer, with unresectable, recurrent, or metastatic disease. Patients with ampullary tumours were also included. They needed to have measurable disease on CT/MRI per RECIST 1.1, an ECOG performance status of 0 or 1, be at least 18 years old with a life expectancy of more than three months and met adequate organ function and laboratory parameters. Availability of tumour tissue and blood samples for biobanking was required, along with compliance with contraceptive measures and pregnancy testing for women of childbearing potential. Exclusion criteria included prior chemotherapy for aBTC, active infections or autoimmune diseases requiring systemic treatment, prior exposure to anti-PD-1/PD-L1 or anti-CTLA4 agents, unresolved biliary obstruction, and a history of other malignancies except for specific exceptions.

### Study treatment

Patients received pembrolizumab at a dose of 200 mg intravenously on Day 1 of each 21-day cycle, alongside cisplatin at 25 mg/m² IV on Days 1 and 8, and gemcitabine at 1000 mg/m² IV on Days 1 and 8. Dose adjustments were made as needed to manage toxicity, including hematologic, renal, and neurological adverse effects according to local practice; no dose adjustment of pembrolizumab was planned.

Safety assessment and monitoring included an interim analysis after 10 patients had completed two cycles, regular evaluations for hematologic toxicity, renal function, and immune-related adverse events, as well as audiometry for detecting cisplatin-induced ototoxicity. Patients were followed for two years post-treatment, with assessments conducted every three months. Those who discontinued treatment before documented disease progression continued to be monitored.

Tumour response was evaluated via CT/MRI scans every three months and assessed by RECIST 1.1 for standard response evaluation and iRECIST for immune-related tumour responses. Additionally, PFS, OS, and biomarker analysis were also performed. Treatment continued until confirmed disease progression (including chemotherapy is such an approach was considered appropriate by treating investigator), unacceptable toxicity, or two years from enrolment, whichever happened first. Patients whose disease progressed could receive further treatment at the investigator's discretion.

### Objectives and endpoints

The study aimed to evaluate the impact of adding pembrolizumab to the standard CisGem chemotherapy regimen compared to historical data. The study also sought to assess both short- and long-term patient outcomes and evaluate the safety profile of pembrolizumab in combination with CisGem. Additionally, exploratory objectives included identifying predictive clinical and pathological biomarkers and assessing immune responses through Tcell phenotyping, MDSCs and Monocytes characterization.

The primary endpoint of the study was the PFS rate at six months, using RECIST 1.1 criteria. Secondary endpoints included the PFS rate at six months, using iRECIST criteria, the ORR using RECIST 1.1 and iRECIST, duration of response, OS, and toxicity assessment according to CTCAE v5.0. Exploratory endpoints focused on biomarker evaluations and immunological response assessments.

### Statistical analysis

The primary objective was to detect an increase in PFS rate at 6 months based on RECIST 1.1 assessment.

A total of 47 evaluable patients were required to identify an improvement in 6mPFS-rate from 60% (null hypothesis) to 75% (alternative hypothesis), using the A'Hern design with a one-sided type I error of 10% and 80% power. A 5% drop out was assumed, thus the study planned to enrol 50 patients. According to the study design, if the lower bound of the two-sided 80% CI of the 6mPFS-rate was above 60%, the combination would be considered active.

The trial, which was non-randomized, had an estimated accrual rate of six patients per month, with a total study duration projected at 36 months, including follow-up. Two key statistical analyses were planned: one at six months post-last patient enrolment for primary PFS assessment and another after two years for long-term PFS and OS.

The following analysis populations were used for the analysis of the study: per protocol population was utilised for efficacy outcomes, included all patients who were assessed evaluable for efficacy per medical review and started protocol treatment (at least one dose of each study drug); the safety population included all patients who started protocol treatment (at least one dose of each study drug) and was utilised to report on toxicity and safety. PFS and OS curves were estimated using the Kaplan-Meier method. Medians were displayed with their two-sided 95% confidence intervals. The PFS rate at 6 months was estimated using Kaplan-Meier estimate at the time point of interest together with the two-sided 80% confidence interval (CI).

Patients alive and event free prior to the analysis cut-off date were censored at the date of the latest assessment. Safety was evaluated using CTCAE version 5.0. The worst grade per patient and per CTCAE term over the study period was tabulated. Subgroup analyses explored intra-hepatic vs. extra-hepatic/gallbladder cancers.

### Translational research

Access to tumour tissue at baseline, and research tumour biopsy and blood sample acquisition at week 6–9 from time of treatment initiation for TR was required. The objectives of the translational research analysis included identification of molecular and immunological characteristics in tumour tissue that could be associated with response to treatment at treatment start or early after treatment initiation, description of PFS and OS according to baseline PD-L1 and MSI, to evaluate if the immune status of the tumour could be determined via circulating immune cell populations and the analysis of the absolute and relative changes in a set of immune-related factors in response to treatment. These analyses were performed in the per protocol population having available samples and data. All analyses were performed with the aim of hypotheses generation only.

#### Immunohistochemistry (PD-L1, MMR testing)

Immunohistochemistry (IHC) was carried out according to the manufacturer’s instructions to assess the expression of PD-L1 (clone 22-C3, Agilent, Santa Clara, USA) in 28/50 patients’ primary tumour samples. The staining was evaluated for the Combined Positive Score (CPS), as described by the manufacturer guidelines (Agilent). For MMR assessment, we applied a stepwise testing algorithm. PMS2 and MSH6 (clone EP51 and EP49, respectively, Agilent, Santa Clara, USA) were stained first; in case of aberrant expression, additional staining for MLH1 or MSH2 was performed. Deficient (d)MMR was defined as concordant loss of MLH1/PMS2 or MSH2/MSH6, or isolated loss of PMS2 or MSH6. Proficient (p)MMR was defined as retained nuclear expression of PMS2 and MSH6 [15].

#### Immune cell subsets

Peripheral Blood Mononuclear Cells (PBMCs) were centrally collected and isolated at the Integrated BioBank of Luxembourg (IBBL). Fluorescence-activated cell sorting (FACS) staining for PBMCs were performed with specific antibodies (detailed in **Supplementary Table 1**) as recommended by the manufacturer’s data sheets. The subpopulations were analyzed on a FACSCanto II flow cytometry system (BD Biosciences). Fluorescence was measured with a minimum of 100,000 events per sample. Data analysis was performed using DIVA software and FlowJo (BD Biosciences).

The NLR (Neutrophil-Lymphocyte-Ratio) was calculated by dividing the absolute neutrophil count by the absolute lymphocyte count at baseline, before the first cycle of immunotherapy, and at EOT. A “normal NLR” is defined as under 3. Higher levels are associated with inflammation, infection and immune system imbalance. The change from baseline to EOT is shown as ΔNLR. ΔNLR <1 represents a clear decrease and >1 an increase of NLR.

No formal adjustment for multiple testing was applied, as the biomarker analyses were considered exploratory due to the sample size; all analysis are seen as hypothesis-generating.

### Trial conduct

The trial commenced recruitment in January 2020 across 7 sites in 3 countries (United Kingdom, Germany and Spain). During the study conduct, a total of eight protocol amendments (last one approved 16th December 2022) were implemented. The study was approved by ethics committee and registration number with EudraCT (EudraCT 2017–003,323-30) and ClinicalTrials.gov (NCT03260712). The study was sponsored by the European Organisation for Research and Treatment of Cancer (EORTC) and coordinated by the EORTC Gastrointestinal Tract Cancer Group. The study was an academic study, funded by MSD, who had no input in the study design and interpretation of results.

## Results

### Patient characteristics

The study enrolled a total of 50 patients between January 2020 and April 2021. Patient characteristics of 50 patients recruited are summarised in Table 1. The median age of participants was 63 years, ranging from 35 to 84 years. The majority of patients were female (62%) and had ECOG-PS of 0 (64.0%) at the time of study entry. Primary tumours were iCCA (60.0%), GBC (22.0%), hCCA (6.0%), dCCA (4.0%) and ampullary tumours (4.0%). Two patients (4.0%) had both intra and extrahepatic cholangiocarcinoma. The majority of patients had stage IV disease (66.0%), and 42% were diagnosed de novo at advanced stage. Four patients (8.0%) had prior curative surgery and two (4.0%) had received adjuvant therapy (none of them with CisGem). Two (4.0%) also had radiotherapy for localised disease.

**Table 1:** Patient characteristics.

Variable	Category	CisGem + Pembrolizumab (n = 50)
		n (%)
Gender	Male	19 (38.0)
	Female	31 (62.0)
Age at registration	Median (range)	63.0 (35.0–84.0)
ECOG performance status	0	32 (64.0)
	1	18 (36.0)
Primary site	Intrahepatic cholangiocarcinoma	30 (60.0)
	Perihilar cholangiocarcinoma	3 (6.0)
	Distal cholangiocarcinoma	2 (4.0)
	Gallbladder cancer	11 (22.0)
	Ampulla of Vater cancer	2 (4.0)
	Both intra- and extra-hepatic biliary tract cancer	2 (4.0)
Histological grade at initial diagnosis	Missing	22 (44.0)
	G1	3 (6.0)
	G2	13 (26.0)
	G3	12 (24.0)
Plasma CEA (μg/L or ng/mL)	Median (Q1-Q3)	3.0 (1.7–11.0)
CA 19.9 (kU/L or IU/mL)	Median (Q1-Q3)	113.5 (20.5–601.0)
CA 125 (kU/L or IU/mL)	Median (Q1-Q3)	35.5 (13.0–199.5)
AJCC stage study entry	Stage II	1 (2.0)
	Stage IIIA	1 (2.0)
	Stage IIIB	9 (18.0)
	Stage IV	33 (66.0)
	Stage IVB	5 (10.0)
	Missing	1 (2.0)
Stage of biliary tract cancer	Non-resectable biliary tract cancer	21 (42.0)
	Recurrent/metastatic biliary tract cancer	18 (36.0)
	Missing	11 (22.0)
Prior curative surgery for disease under study	No	46 (92.0)
	Yes	4 (8.0)
Prior adjuvant therapy	No	48 (96.0)
	Yes	2 (4.0)
Prior radiotherapy for localised disease	No	48 (96.0)
	Yes	2 (4.0)

Abbreviations: n: number, CisGem: Cisplatin/Gemcitabine; G: grading.

### Treatment received

All patients (n = 50) started the allocated treatment of the experimental arm (cisplatin, gemcitabine, and pembrolizumab). The median treatment duration was 34.5 weeks, with a range between 4.0 and 110.4 weeks. The median number of treatment cycles administered was 11 (range 1.0–35.0). Delays in treatment were common, with 35 patients (70%) experiencing at least one pembrolizumab dose delay, 31 patients (62%) encountering at least one cisplatin dose delay, and 38 patients (76%) undergoing at least one gemcitabine dose delay. Dose reductions were implemented in 13 patients (26.0%) for cisplatin and 22 patients (44.0%) for gemcitabine. Gemcitabine dose was re-escalated in 7 patients. Relative dose intensity was as follows: median 90.6% (Q1-Q3 79.2–95.5), 54.4% (Q1-Q3 42.9–75.0) and 72.3% (Q1-Q3 46.3–82.2) for pembrolizumab, cisplatin and gemcitabine, respectively.

### Primary endpoint

At the time of primary endpoint analysis, 44 patients had progressed and 37 had died; all patients had discontinued treatment, 32 (64.0%) due to disease progression, 3 (6.0%) due to pembrolizumab-related AEs, 3 (6.0%) due to patient/clinician decision, 2 (4.0%) due to death not due to malignant disease or toxicity, 1 (2.0%) due to other malignancy and 6 (12%) due to other reasons (one of whom died due to biliary sepsis). Three (6.0%) stopped due to completion of the pre-planned 2 years of therapy.

The primary endpoint was evaluated in the per-protocol population, which accounted for 49 eligible patients who started CisGem and pembrolizumab (CONSORT diagram provided in **Supplementary Figure 1**). One patient was deemed ineligible due to not meeting one of the eligibility criteria (high CRP) and was therefore excluded from the efficacy analysis. Median follow-up at the time of the PFS evaluation was 26.1 months. PFS rate at 6-months (the primary endpoint) was 61.2% (80% CI 51.7–69.5) Given that the lower bound of the two-sided 80% CI is below 60% the study did not meet its primary endpoint. All patients had available PFS data at 6 months; therefore, the sensitivity analyses foreseen to verify the robustness of the results adjusted for missing data was not performed.

### Secondary objectives

Results for efficacy-related secondary objectives are summarised in Table 2. The estimated mPFS was 8.3 months (95% CI 5.6 -10.6) (**Supplementary Figure 2A**). The ORR was 40.8% (95% CI 27.0–55.8) with a median duration of response of 10.1 months (95% CI 4.9-not reached). Eighteen patients (36.7%) achieved disease stabilisation as their best response with a median duration of disease stabilisation of 10.6 months (95% CI 8.3 -13.6). Response and progression outcomes assessed by iRECIST were comparable, with no major discrepancies (Table 2). The estimated mOS was 13.4 months (95% CI 8.3 -20.5) (**Supplementary Figure 2B**), with an OS-rate at 24 months of 28.4% (95% CI 16.6–41.4).

**Table 2:** Efficacy outcomes.

Outcome		CisGem + Pembrolizumab (n = 50)
Progression-free survival (RECIST 1.1)	6-month PFS rate (80% CI)	61.2% (51.7–69.4)
	Median (95% CI)	8.28 months (5.59–10.58)
	6-month PFS rate (95% CI)	61.2% (46.2–73.2)
	12-month PFS rate (95% CI)	30.6% (18.4–43.6)
	18-month PFS rate (95% CI)	16.3% (7.6–27.9)
	24-month PFS rate (95% CI)	12.2% (5.0–23.0)
Progression-free survival (iRECIST 1.1)	6-month PFS rate (80% CI)	63.3% (53.7–71.4)
	Median (95% CI)	8.34 months (5.62–11.10)
	6-month PFS rate (95% CI)	63.3% (48.2–75.0)
	12-month PFS rate (95% CI)	32.7% (20.1–45.8)
	18-month PFS rate (95% CI)	16.3% (7.6–27.9)
	24-month PFS rate (95% CI)	12.2% (5.0–23.0)
Overall survival	Median (95% CI)	13.37 months (8.28–20.53)
	6-month OS rate (95% CI)	81.6% (67.7–90.0)
	12-month OS rate (95% CI)	53.1% (38.3–65.8)
	18-month OS rate (95% CI)	38.8% (25.3–52.0)
	24-month OS rate (95% CI)	28.4% (16.6–41.4)
Best Overall Response Rate (RECIST 1.1)	Overall response rate (95% CI)	40.8% (27.0–55.8)
	Duration of response (estimated median (95% CI))	10.09 months (4.90-not reached)
	Partial response (PR) (n (%))	20 (40.8%)
	Stable disease (SD) (n (%))	18 (36.7%)
	Duration of disease estabilisation (estimated median (95% CI))	10.55 months (8.25–13.60)
	Progressive disease (PD) (n (%))	6 (12.2%)
	Early death (n (%))	5 (10.2%)
Best Overall Response Rate (iRECIST 1.1)	Overall response rate (95% CI)	40.8% (27.0–55.8)
	Duration of response (estimated median (95% CI))	9.54 months (4.90–11.56)
	Partial response (PR) (n (%))	20 (40.8%)
	Stable disease (SD) (n (%))	18 (36.7%)
	Duration of disease stabilisation (estimated median (95% CI))	10.96 months (8.28–13.60)
	Progressive disease (PD) (n (%))	6 (12.2%)
	Early death (n (%))	5 (10.2%)

Abbreviations: n: number, CisGem: Cisplatin/Gemcitabine; PFS: progression-free survival; OS: overall survival; PR: partial response; SD: stable disease; PD: progressive disease; HR: hazard ratio; CI: confidence interval.

In terms of safety and adverse events (**Supplementary Table 2**), 76.0% of patients had a grade 3 or greater treatment-related AEs. The most frequently observed grade 3–5 toxicities included hematologic effects such as neutropenia in 30% of patients and anaemia in 24%. Thrombocytopenia was reported in 16% of patients. Grade ≥3 fatigue was another common adverse event, affecting 12% of participants. There were two treatment related deaths: one due to hypoxia and one due to acute kidney injury.

### Translational research

A total of 28 primary tumours samples were available for translational research. Tumour tissue and blood samples (22/50) were collected for characterization of T-cells, monocytes, MDSCs, and PD-1/PD-L1 at baseline and week 6–9 by FACS. The analysis of these immune cell subsets should provide insight into their prognostic relevance before and during anti-PD-L1 therapy in aBTC. Furthermore, PD-L1 staining (clone 22-C3) was evaluated for CPS in 28/50 patients to clarify the dependence on survival data.

#### Identification of molecular and immunological characteristics associated with outcomes

**Fig. 1:**
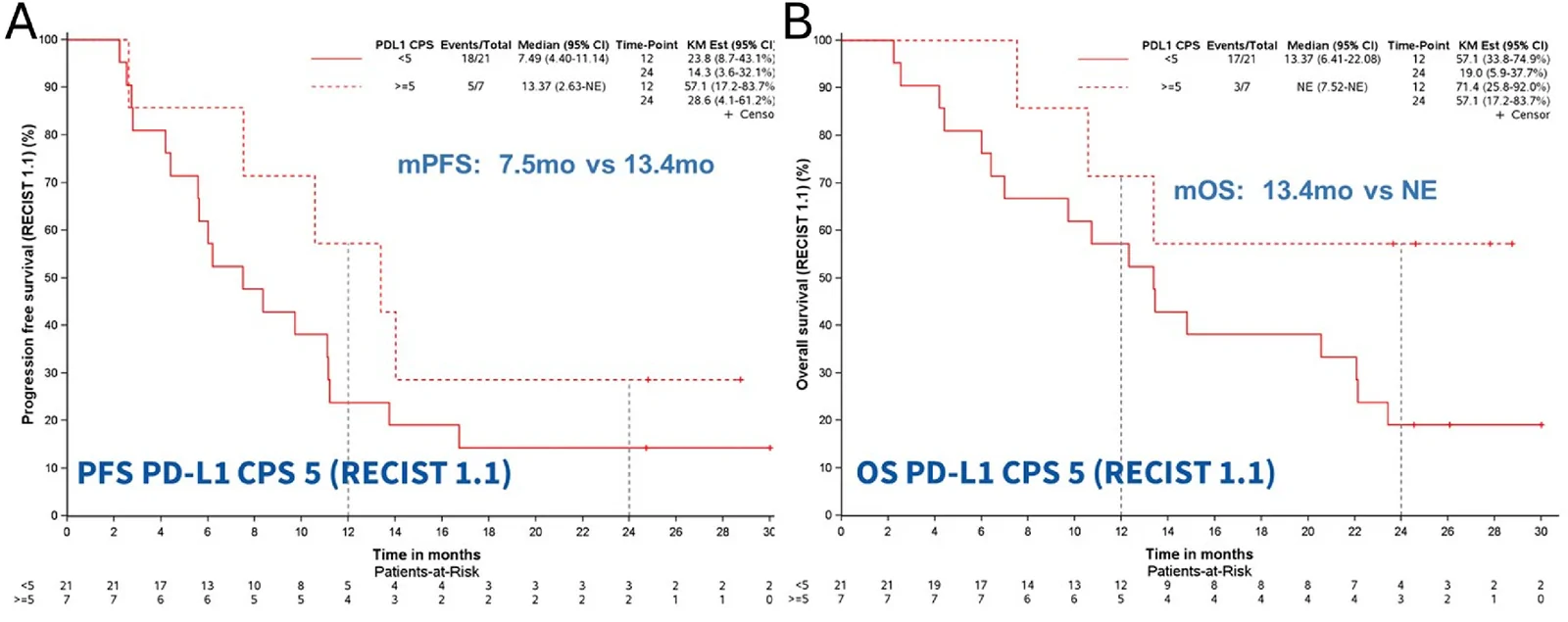
Outcomes by PD-L1 expression at baseline (CPS 5), A) Progression-free survival and B) Overall survival.

Abbreviations: n: number; (m)PFS: (median) progression-free survival; (m)OS: (median) overall survival; CI: confidence interval; CPS: combined positive score; mo: month; HR: hazard ratio; CI: confidence interval; NE: Not Estimable.

All tested patients (27/50) were pMMR. In the final analysis, PFS and OS were longer depending on PD-L1 when the CPS ≥1 cut-off was utilized (Table 3), but not significant. Findings were similar with CPS ≥5 cut-off (Table 3**;** Fig. 1).

**Table 3:** Outcomes by PD-L1 expression at baseline (CPS 1 and CPS 5).

	PFS				OS			
PD-L1 CPS n = 28	Event/ Total	Median (95% CI)^1^	Hazard Ratio (95% CI)^2^	P-value	Event/ Total	Median (95% CI)^1^	Hazard Ratio (95% CI)^2^	P-value
**<1**	10/11	**6.21** (2.76–11.14)	1.69 (0.73–3.91)	0.2146^3^	9/11	**12.32** (4.21–23.43)	1.48 (0.61–3.58)	0.3833^3^
**≥1**	13/17	**10.58** (4.40–16.72)	Reference		11/17	**13.44** (9.72-NE)	Reference	
**<5**	18/21	**7.49** (4.40–11.14)	1.76 (0.65–4.77)	0.2593^3^	17/21	**13.37** (6.41–22.08)	2.60 (0.76–8.90)	0.1136^3^
**≥5**	5/7	**13.37** (2.63-NE)	Reference		3/7	**NE** (7.52-NE)	Reference	

Abbreviations: n: number; PFS: progression-free survival; OS: overall survival; CI: confidence interval; CPS: combined positive score; HR: hazard ratio; CI: confidence interval;^1^Kaplan-Meier method; ^2^Cox model; ^3^Logrank test.

#### Analysis of the absolute and relative changes in a set of immune-related factors in response to treatment

**Fig. 2:**
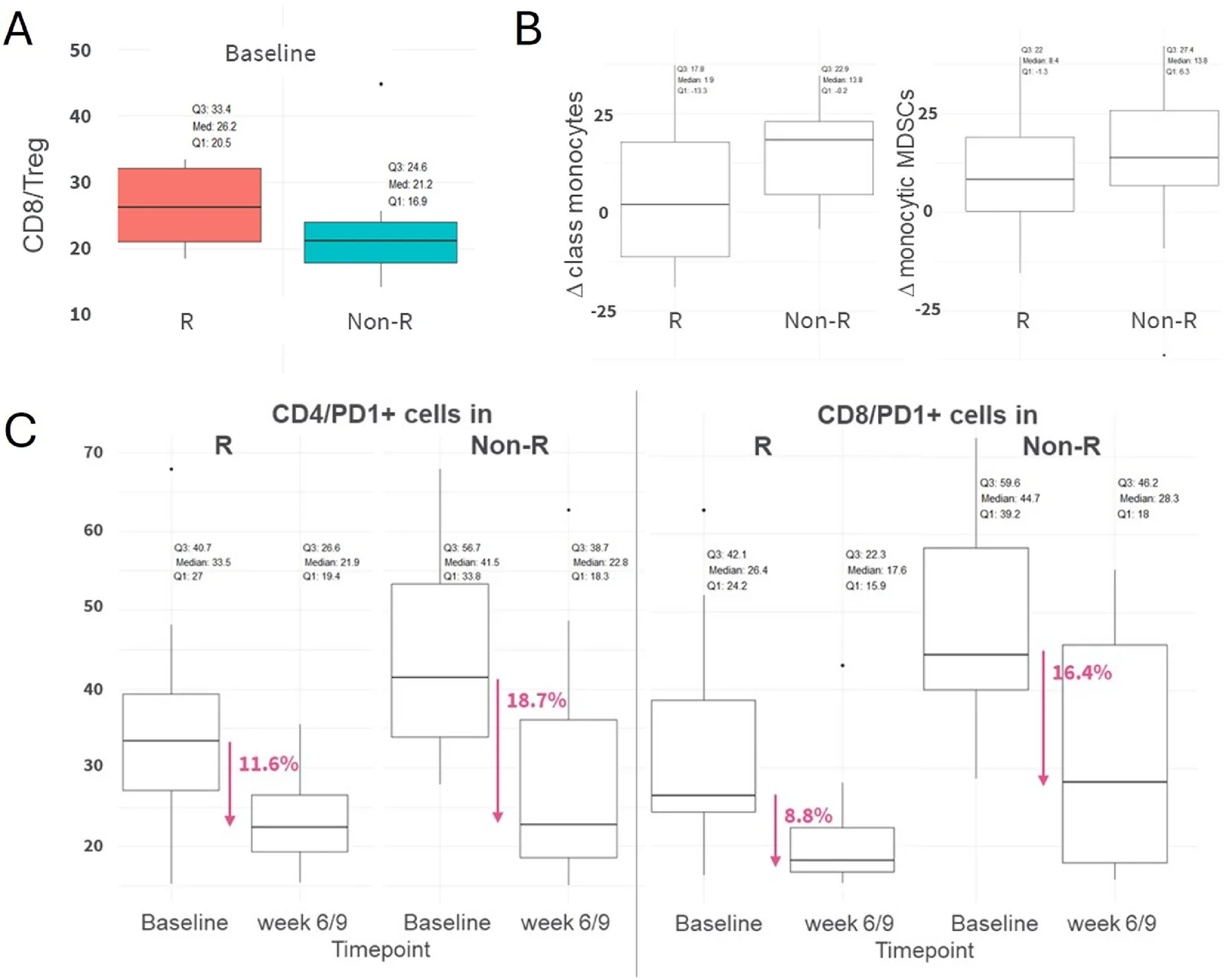
Characterization of immune cells,A)CD8/Treg ratio at baseline; B) CD4/PD1+ and CD8/PD1+ Tcells during treatment and C) Median Variations in monocytes over time.

Abbreviations: R: Responder; Non-R: Non-responder; Treg: regulatory T cell; MDSC: myeloid-derived suppressor cell.

The immune cell subset analysis focused on CD4 and CD8 T cells, depending on their PD-1 status and on MDSCs, showing that Responders (n = 10; defined as patients achieving RECIST 1.1-defined complete or partial response) showed a higher baseline median CD8/Treg ratio than Non-Responders (n = 12, 26.2% vs 21.2%) (Fig. 2A). This effect was lost after 9 weeks of treatment (19.1%vs 19.8%).

In 22/50 PBMC samples, no changes were seen in median % of total CD4-, CD8- and Treg-cells during treatment (baseline, week 6/9). Median % of CD4 and CD8 cells were higher in Responders (n = 10) than in Non-Responders (n = 12) (20%vs 14% and 8% vs 6%, respectively).

There was a decrease in median % of CD4/PD1+ and CD8/PD1+ cells during treatment (36.0% vs 22.2% and 41.6% vs 21.0%, respectively) in all patients. Responders had lower median % of CD4/PD1+ and CD8/PD1+ cells at baseline vs Non-Responders (33.5% vs 41.5% and 26.4% vs 44.7%, respectively). A similar decrease in median % of CD4/PD1+ and CD8/PD1+ cells (-11.6% vs -18.7% and -8.8% vs -16.7%, respectively) were seen in Non-Responders and Responders (Fig. 2B).

In the Non-Responders vs Responders group from baseline to week 6/9 (Δ), a higher median increase of classical monocytes (13.8% vs 1.9%) and of MDSCs (13.8% vs 8.4%) was identified in the Non-Responder group (Fig. 2C).

#### NLR

While NLR >3 is classified as signal for inflammation, infection and immune system imbalance, Non-Responders (n = 30) constantly had elevated median NLR levels (>3) versus Responders (NLR<3, n = 20) at both baseline and EOT (3.7 vs. 2.7 and 4.3 vs. 2.7, respectively). Median NLR remained unchanged across the cohort of all patients.

Furthermore, high stage (IV) patients showed increased median NLR levels versus lower stage (II/III) patients at start and end of treatment, but the increase at EOT was higher in the low stage cancer patients (ΔmNLR 1.2 vs 0.4).

Among patients tested for CPS (28/50), all had comparable median NLR<3 at treatment start. CPS-low patients, irrespective of CPS <1 (n = 11) or <5 (n = 21) showed nearly a median NLR doubling until EOT (ΔmNLR∼2). In contrast, clearly CPS-positive patients, defined as CPS >1 (n = 17) or >5 (n = 7), exhibited a median NLR decrease during treatment (ΔmNLR<1), with values remaining median NLR< 3 at EOT (**Supplementary Table 3 and Supplementary Figure 3**).

## Discussion

The EORTC-1607-GITCG/ABC-09 trial was designed before any already published randomized phase -III trials to evaluate whether the addition of pembrolizumab to standard chemotherapy (CisGem) could provide enhanced efficacy while enabling exploratory biomarker analysis to better characterise responders. Although the study did not meet its primary endpoint of improving 6-month PFS beyond the predefined threshold (observed rate: 61.2%; 80% CI: 51.7–69.5), the results are in line with the benchmark set by prior randomized studies in this space [4].

The trial's secondary outcomes, including mPFS of 8.3 months and mOS of 13.4 months, compare favourably to those reported in the phase III TOPAZ-1 and KEYNOTE-966 trials [3,4]. In TOPAZ-1, durvalumab combined with gemcitabine and cisplatin improved median OS to 12.8 months compared with 11.5 months in the placebo arm (HR 0.80), with a median PFS of 7.2 months. Similarly, KEYNOTE-966 reported a mOS of 12.7 months with pembrolizumab, although mPFS improvement (6.5 vs 5.6 months) did not reach statistical significance. Notably, the ORR observed in our trial (40.8%) was numerically higher than those reported in both phase III studies (26.7% in TOPAZ-1 and 29% in KEYNOTE-966), albeit in a single-arm phase II setting with smaller number of patients and, consequently, large 95%-confidence intervals. Fully aware that cross-trial comparisons are exploratory, based on the mechanism of PD-(L)1 inhibition, ORR discrepancies may arise from differences in the patient population, eligibility criteria (e.g., tumour location or recurrence time), different endpoints and outcome definitions, or therapy duration. The different patient population, eligibility criteria, e.g., tumour locations or recurrence time, different endpoints and outcome definitions, or therapy duration, must be borne in mind.

These results support the continued exploration of immune-checkpoint inhibitors in BTC, particularly in combination with chemotherapy with CisGem backbone. The fact that benefit is seen across broad patient populations with only a minority achieving durable responses reinforces the need for robust biomarkers [13]. Prior efforts to stratify patients based on PD-L1 expression in BTC have yielded disappointing results. Both placebo-controlled trails: TOPAZ-1 (using TAP score, with Ventana, SP263) and KEYNOTE-966 (using CPS score with Agilent, 22C3) failed to demonstrate a predictive role for PD-L1, with similar hazard ratios for survival observed regardless of PD-L1 status. All tumours tested were microsatellite stable, reflecting the low frequency of MSI high status in BTC. During the trial, no additional information regarding key genomic alterations such as IDH1 mutation, FGFR2 fusion, HER2 amplification, BRAF V600E could be received due to missing informed consent or reimbursement.

The translational research component of this study offers important insights, however results should interpreted as hypothesis-generating. Due to the limited sample size an increased risk of false-positive findings is possible, nevertheless, these signals warrant further investigation. While bulk immune phenotyping of peripheral blood did not show major changes in CD4+, CD8+, or Treg cell populations during treatment, subtle differences were observed between Responders and Non-Responders. At baseline, Responders exhibited a higher CD8/Treg ratio, a finding that was not sustained at week 6–9, suggesting that early immune contexture may be more relevant than on-treatment changes [16]. The higher CD8/Treg ratio indicates fewer amount of Tregs which is accompanied by an enhanced T cell response similar shown by Guo et al. for aBTC [17]. Furthermore, lower levels of PD1+ expression on both CD4+ and CD8+ cells at baseline were noted in Responders compared to Non-Responders. These findings hint at an exhausted T-cell phenotype potentially being a negative predictive marker [18] or, alternatively, a prognostic factor. Additionally, under anti-PD-1 treatment we found a reduction of these cells in all patients.

Interestingly, a higher increase in MDSCs and classical monocytes was observed in Non-Responders compared to Responders, suggesting a potential immunosuppressive shift in these patients. The role of MDSCs to increase tumour aggressiveness in BTC [19] and induce resistance to immunotherapy is increasingly recognized [20], and these findings warrant further investigation in larger datasets, given the small size of our cohort and the potential statistical limitations. Similarly, a higher number of monocytes can induce Tcell paralysis and tumour-mediated immunosuppression leading to resistance to immune checkpoint [21]. The same mechanism is known from other cancer entities like NSCLC [22] and melanoma [23], again associated with poor prognosis and shortening survival time.

The neutrophil-to-lymphocyte ratio (NLR) reflects the balance between pro-tumoral inflammation and anti-tumoral immune responses [24,25]. An NLR >3 indicates chronic inflammation and immune system imbalance. In the context of ICI therapy, Non-Responders often show a limited anti-tumour response driven by pro-tumour inflammation. Consequently, elevated higher NLR levels are associated with poorer OS, PFS, and reduced clinical benefit across various cancer types [26]. As here seen in small numbers, Responders, low stage and CPS positive patients are characterised by a normal NLR. The opponent groups showed an enhanced NLR and an additional increase during ICI treatment. Unfortunately, our low patient numbers showed no correlation for NLR and survival times. In upper GI cancers, low NLR values predict better efficacy of ICIs and improved survival [27,28] as well as in advanced disease stages of lung cancer [29]. The combination of PD-L1 and NLR may be a simple and promising prognostic indicator [30].

While exploratory and hypothesis-generating, these biomarker analyses are a step toward more personalized use of immunotherapy in BTC; this may be of special interest in the second-line setting, to identify patients who may still benefit from maintaining a chemo-immunotherapy strategy after progression to first-line treatment. Our results highlight the importance of understanding the tumour microenvironment and peripheral immune landscape to identify patients most likely to benefit from immune checkpoint blockade. Furthermore, the limited predictive value of PD-L1 expression underlines the need for more sophisticated composite biomarkers, potentially integrating genomic, immunologic, microbiome and inflammatory parameters.

This study has several strengths, including prospective biospecimen collection, centralized immune profiling, and a well-defined treatment regimen. However, limitations for generalizability of these findings such as relatively small sample size, some missing biospecimens, and the lack of validation cohort must be acknowledged. As a single-arm phase II trial, comparisons to historical controls are limited. In addition, samples for translational research were available for not all patients; taking into account the real challenges with tissue acquisition in BTC.

In conclusion, the EORTC-1607-GITCG/ABC-09 study confirmed the activity of pembrolizumab in combination with chemotherapy for patients with advanced BTC, with efficacy outcomes comparable with the larger randomized trials of ICIs with a similar mechanism of action. Importantly, the exploratory biomarker analyses offer promising avenues for identifying immune phenotypes associated with response and resistance. These findings should serve as the foundation for future prospective biomarker-driven studies in this setting.

## Registration number

EudraCT 2017-003,323-30; NCT03260712.

## Funding

Supported in part by a research grant from Investigator-Initiated Studies Program of Merck Sharp & Dohme Corp. The opinions expressed in this paper are those of the authors and do not necessarily represent those of Merk Sharp & Dohme Corps. Dr Angela Lamarca was funded by a SEOM (Spanish society of Medical Oncology) return fellowship. Translational research analyses were supported by the EORTC Gastrointestinal Tract Cancer Group (GITCG) and self-funded by Prof. Moehler, University Medical Center of the Johannes Gutenberg University Mainz.

## CRediT authorship contribution statement

**Angela Lamarca:** Writing – original draft, Visualization, Project administration, Methodology, Funding acquisition, Data curation, Conceptualization. **Annett Maderer:** Writing – original draft, Visualization, Validation, Methodology. **Daniel C. Wagner:** Methodology, Data curation. **Mairéad G. McNamara:** Validation, Investigation. **John A. Bridgewater:** Validation, Investigation, Conceptualization. **Arvind Arora:** Validation, Investigation. **Teresa Macarulla:** Validation, Investigation. **Jorge Adeva:** Validation, Investigation. **Richard A. Hubner:** Validation, Investigation. **Florian Lordick:** Validation, Investigation. **Friedrich Foerster:** Validation, Investigation. **Arndt Weinmann:** Validation, Investigation. **Peter R. Galle:** Validation, Investigation. **Bryan Leurquin:** Writing – original draft, Visualization, Validation, Methodology, Formal analysis, Data curation. **Sandrine I. Marreaud:** Validation, Conceptualization. **Murielle E. Mauer:** Writing – original draft, Validation, Software, Methodology, Formal analysis, Data curation, Conceptualization. **Juan W. Valle:** Writing – original draft, Validation, Project administration, Methodology, Conceptualization. **Markus Moehler:** Writing – original draft, Validation, Project administration, Methodology, Investigation, Data curation, Conceptualization.

## Declaration of competing interest

The authors declare the following financial interests/personal relationships which may be considered as potential competing interests:

Angela Lamarca declares Travel and educational support from Ipsen, Pfizer, Bayer, AAA, SirtEx, Novartis, Mylan, Delcath Advanz Pharma and Roche; Speaker honoraria from Merck, Pfizer, Ipsen, Incyte, AAA/Novartis, QED, Servier, Astra Zeneca, EISAI, Roche, Advanz Pharma and MSD; Advisory and consultancy honoraria from EISAI, Nutricia, Ipsen, QED, Roche, Servier, Boston Scientific, Albireo Pharma, AstraZeneca, Boehringer Ingelheim, GENFIT, TransThera Biosciences, Taiho, MSD and Viatris; Principal Investigator-associated Institutional Funding form QED, Merck, Boehringer Ingelheim, Servier, Astra Zeneca, GenFit, Panbela Therapeutics, Novocure GmbH, Camurus AB, Albireo Pharma, Taiho, TransThera, Jazz Therapeutics and Roch; Member of the Knowledge Network and NETConnect Initiatives funded by Ipsen.

**Juan W Valle** declares Honoraria/Advisory Boards (personal): AstraZeneca, Incyte, Taiho Oncology, Servier, and Jazz Pharmaceuticals. Honoraria/Advisory Boards (institution): Redx Pharma, Cogent Biosciences, Owkin, Jazz Pharmaceuticals, and Oncosil.

**Markus Moehler** declares Honoraria (invited Speaker (IS))/Advisory Boards (AB): AIO (IS), Amgen (AB), Amgen (IS), Astellas (IS), Astellas (AB),Astra-Zeneca (IS), Astra-Zeneca (AB),Bayer (AB), Beigene (IS), Beigene (AB), BMS (AB), BMS (IS), Daiichi (AB), Daiichi (IS), EORTC, Other, Personal, ChairRG communications (IS), Lilly (AB),MSD (AB), MSD (IS), Novartis (AB),Sanofi, Expert Testimony, Servier (AB) and Taiho (AB).

Other co-authors declared no conflict of interest.

## Appendix Supplementary materials

Supplementary Figure 1: CONSORT diagram Image, image 1 dummy alt text

Supplementary Figure 2: A) Progression-free survival (RECIST 1.1) and B) Overall survival Image, image 2 dummy alt text

Supplementary Figure 3: Changes in NLR, A) NLR changes from baseline to EOT in Responders and Non-Responders; B) NLR changes from baseline to EOT in CPS low (<1 and >5) and C) high (>1 and >5) patients. Abbreviations: NLR: Neutrophil-Lymphocyte-Ratio; EOT: end of treatment; CPS: combined positive score. Image, image 3 dummy alt text

Image, application 4

Image, application 5

Image, application 6
