# Supplementary material for: EORTC-1607-GITCG/ABC-09: Open-label first-line, single-arm phase II study of CisGem combined with pembrolizumab in patients with advanced or metastatic biliary tract cancer
Source: Transl Oncol. 2026 Aug 31;72:103003. doi: 10.1016/j.tranon.2026.103003 (PMC13563234; doi:10.1016/j.tranon.2026.103003)
Supplement: Supplementary file 1 [file mmc4.docx]

## Supplementary Table 1: Antibody declaration (all antibodies from BD Biosciences, San Jose, CA, USA)

| Antigen | Fluorochrom | Clone | Best.Nr. |
| --- | --- | --- | --- |
| CD3 | V500 | UCHT1 | 561416 |
| CD4 | V450 | RPA-T4 | 561838 |
| CD8 | PerCP | SK1 | 345774 |
| CD11b | APC | ICRF44 | 550019 |
| CD14 | PeCy7 | M5E2 | 557742 |
| CD15 | V500 | HI98 | 561585 |
| CD16 | V450 | 3G8 | 644489 |
| CD25 | PeCy7 | 2A3 | 335824 |
| CD33 | PE | P67.6 | 345799 |
| CD45 | APC-H7 | 2D1 | 560178 |
| CD127 | FITC | HIL-7R-M21 | 560549 |
| CD152 | APC | BNI3 | 555855 |
| HLA-DR | PerCP | L243 | 347402 |
| PD-1 | PE | EH12.1 | 560795 |
| PD-L1 | FITC | MIH1 | 558065 |
