# Supplementary material for: EORTC-1607-GITCG/ABC-09: Open-label first-line, single-arm phase II study of CisGem combined with pembrolizumab in patients with advanced or metastatic biliary tract cancer
Source: Transl Oncol. 2026 Aug 31;72:103003. doi: 10.1016/j.tranon.2026.103003 (PMC13563234; doi:10.1016/j.tranon.2026.103003)
Supplement: Supplementary file 2 [file mmc5.docx]

## Supplementary Table 2: Treatment-related adverse events

This table shows the number of patients by worst grade reported for each preferred term.

| **Treatment related adverse event (grade 3-5) (n=50)** | **Grade 1 n (%)** | **Grade 2 n (%)** | **Grade 3 n (%)** | **Grade 4 n (%)** | **Grade 5 n (%)** | **Grade ≥3 n (%)** |
| --- | --- | --- | --- | --- | --- | --- |
| PATIENTS' WORST GRADE | 2 (4.0) | 5 (10.0) | 23 (46.0) | 13 (26.0) | 2 (4.0) | 38 (76.0) |
| Neutrophil Count Decreased | 2 (4) | 7 (14) | 7 (14) | 8 (16) |  | 15 (30) |
| Anaemia | 1 (2) | 14 (28) | 11 (22) | 1 (2) |  | 12 (24) |
| Platelet Count Decreased | 5 (10) | 3 (6) | 1 (2) | 7 (14) |  | 8 (16) |
| Fatigue | 13 (26) | 14 (28) | 6 (12) |  |  | 6 (12) |
| Abdominal Pain | 3 (6) | 1 (2) | 2 (4) |  |  | 2 (4) |
| Fever | 1 (2) | 1 (2) | 1 (2) | 1 (2) |  | 2 (4) |
| Lung Infection |  | 1 (2) | 2 (4) |  |  | 2 (4) |
| Sepsis |  |  | 2 (4) |  |  | 2 (4) |
| Other AE Infection related |  | 2 (4) | 2 (4) |  |  | 2 (4) |
| Alanine Aminotransferase Increased | 5 (10) |  | 2 (4) |  |  | 2 (4) |
| Ggt Increased |  |  | 2 (4) |  |  | 2 (4) |
| Other AE renal and urinary disorders |  |  | 2 (4) |  |  | 2 (4) |
| Other AE respiratory |  |  | 2 (4) |  |  | 2 (4) |
| Hypertension |  |  | 2 (4) |  |  | 2 (4) |
| Febrile Neutropenia |  |  |  | 1 (2) |  | 1 (2) |
| Myocardial Infarction |  |  | 1 (2) |  |  | 1 (2) |
| Supraventricular Tachycardia |  |  |  | 1 (2) |  | 1 (2) |
| Ascites |  |  | 1 (2) |  |  | 1 (2) |
| Cholecystitis |  |  |  |  | 1 (2) | 1 (2) |
| Other AE |  |  |  |  | 1 (2) | 1 (2) |
| Urinary Tract Infection |  |  | 1 (2) |  |  | 1 (2) |
| Aspartate Aminotransferase Increased | 4 (8) | 1 (2) | 1 (2) |  |  | 1 (2) |
| Blood Bilirubin Increased |  |  | 1 (2) |  |  | 1 (2) |
| White Blood Cell Decreased |  |  |  | 1 (2) |  | 1 (2) |
| Anorexia | 4 (8) |  | 1 (2) |  |  | 1 (2) |
| Hypoalbuminemia |  | 1 (2) | 1 (2) |  |  | 1 (2) |
| Hyponatremia |  |  | 1 (2) |  |  | 1 (2) |
| Peripheral Sensory Neuropathy | 6 (12) | 6 (12) | 1 (2) |  |  | 1 (2) |
| Acute Kidney Injury |  |  |  |  | 1 (2) | 1 (2) |
| Hypoxia |  | 1 (2) |  |  | 1 (2) | 1 (2) |

Abbreviations: n: number.
