# Supplementary material for: EORTC-1607-GITCG/ABC-09: Open-label first-line, single-arm phase II study of CisGem combined with pembrolizumab in patients with advanced or metastatic biliary tract cancer
Source: Transl Oncol. 2026 Aug 31;72:103003. doi: 10.1016/j.tranon.2026.103003 (PMC13563234; doi:10.1016/j.tranon.2026.103003)
Supplement: Supplementary file 3 [file mmc6.docx]

## Supplementary Table 3: NLR overview

|  |  |  | **Baseline** | | **EOT** | |  |
| --- | --- | --- | --- | --- | --- | --- | --- |
|  |  | n | **mNLR** | Q1;Q3 | **mNLR** | Q1;Q3 | Δ mNLR |
|  | **all** | **50** | **3.3** | **2.4; 4.3** | **3.6** | **2.5; 6.9** | **0.5** |
|  |  |  |  |  |  |  |  |
| subgroups | Responder | 20 | **2.7** | 2.1; 3.7 | **2.7** | 2.2; 4.4 | 0.5 |
|  | Non-Responder | 30 | **3.7** | 2.7; 5.5 | **4.3** | 3.3; 8.6 | 0.5 |
|  | lower stage (II/III) | 11 | **2.7** | 2.2; 5.7 | **3.1** | 2.6; 6.9 | 1.2 |
|  | higher stage (IV) | 38 | **3.6** | 2.5; 4.3 | **3.9** | 2.4; 6.5 | 0.4 |
|  | CPS<1, negative | 11 | **2.7** | 2.6; 4.2 | **5.0** | 4; 8.1 | 2.2 |
|  | CPS≥1, positive | 17 | **2.9** | 2.2; 4.1 | **2.8** | 1.9; 4.4 | 0.7 |
|  | CPS<5, negative | 21 | **2.7** | 2.6; 4.2 | **4.5** | 3.5; 8.1 | 1.9 |
|  | CPS≥5, positive | 7 | **2.8** | 1.4; 3.5 | **2.6** | 1.9; 2.8 | 0.3 |

Abbreviations: n: number; (m)NLR: (median) Neutrophil-Lymphocyte-Ratio; EOT: end of treatment; CPS: combined positive score.
